# Comprehensive analysis of androgen receptor status in prostate cancer with neuroendocrine differentiation

**DOI:** 10.3389/fonc.2022.955166

**Published:** 2022-08-09

**Authors:** Ruopeng Su, Lei Chen, Zhou Jiang, Minghao Yu, Weiwei Zhang, Zehua Ma, Yiyi Ji, Kai Shen, Zhixiang Xin, Jun Qi, Wei Xue, Qi Wang

**Affiliations:** ^1^ Department of Urology, Ren Ji Hospital, Shanghai Jiao Tong University School of Medicine, Shanghai, China; ^2^ Department of Pathology, Ren Ji Hospital, Shanghai Jiao Tong University School of Medicine, Shanghai, China; ^3^ Department of Urology, Xinhua Hospital, School of Medicine, Shanghai Jiao Tong University, Shanghai, China; ^4^ Shanghai Key Laboratory for Tumor Microenvironment and Inflammation, School of Medicine, Shanghai Jiao Tong University, Shanghai, China

**Keywords:** prostate cancer, androgen receptor, neuroendocrine differentiation, immunohistochemistry, multiplex immunofluorescence

## Abstract

The androgen receptor (AR) signaling is a key contributor to tumorigenesis and the progression of prostate cancer. A subset of patients may develop neuroendocrine (NE) features, resulting in resistance to androgen deprivation therapy and poor prognosis. In this study, we combined immunostaining and bulk and single-cell transcriptome analyses to better characterize the status of AR in prostate cancer with neuroendocrine differentiation. The exploration of online datasets indicated the existence of AR^HIGH^/NE^HIGH^ prostate cancer and revealed that these double-high cases are majorly present in castration-resistant prostate cancer with a less neuroendocrine-transdifferentiated state. We then reviewed 8,194 prostate cancer cases with available immunohistochemistry reports and found 2.3% cases (*n* = 189) that showed at least one of the NE markers (chromogranin A, synaptophysin, and neural cell adhesion molecule 1) being positive in at least 5% of epithelial cells. Within these 189 cases, we observed that 81.0% cases (*n* = 153) showed AR positive and 19.0% (*n* = 36) showed AR negative. Patients with AR loss tumors demonstrated a correlation with adverse clinical stages, indicating a trade-off between AR and advanced disease in neuroendocrine differentiation. Using multiplex immunofluorescence staining, we observed the co-localization of AR and NE markers in prostate cancer cells. In addition, data mining of single-cell transcriptome further confirmed the existence of AR^HIGH^/NE^HIGH^ prostate cancer cells in castration-resistant samples and suggested that AR still exerts its androgen response and anti-apoptotic effect in these double-high cells. Thus, our study provides a better understanding of AR signaling in the cellular plasticity of prostate cancer with neuroendocrine differentiation and allows new insights into the therapeutic development.

## Introduction

Androgen receptor (AR) and AR signaling are critical in the development and progression of prostate cancer (1). Activation of the AR pathway promotes the proliferation and differentiation of prostate epithelial cells while simultaneously acting as an anti-apoptotic factor (2). Androgen deprivation therapy, which aims to inhibit AR production and AR signaling, still functions as the mainstay in the treatment of early-stage prostate cancer (3–5). However, the clinical efficacy of androgen deprivation therapy is short-lived, and the majority of the patients relapse with castration-resistant prostate cancer (CRPC) (3–6). A subset of the treatment-resistant patients develop neuroendocrine (NE) features, an extremely aggressive variant of prostate cancer that poses a current clinical challenge (7, 8).

Prostate cancer with neuroendocrine differentiation is typically defined by the heterogeneous histological expression of several NE markers in at least 5% of epithelial cells, including chromogranin A (CHGA), synaptophysin (SYP), neural cell adhesion molecule 1 (NCAM1), and enolase 2 (ENO2) (9). WHO 2016 classified prostate cancer with neuroendocrine differentiation morphologically into six categories: (1) usual prostate adenocarcinoma with neuroendocrine differentiation, (2) adenocarcinoma with Paneth cell neuroendocrine differentiation, (3) carcinoid tumor, (4) small cell carcinoma, (V) large cell neuroendocrine carcinoma, and (6) mixed (small or large cells) neuroendocrine carcinoma–acinar adenocarcinoma (9). Most patients with neuroendocrine differentiation have a high rate of visceral metastasis, which is associated with rapid disease progression and poor prognosis (10–13).

An increasing number of studies have investigated the clinicopathology and molecular character of prostate cancer with neuroendocrine differentiation in the past years, but the understanding of AR status in prostate cancer neuroendocrine differentiation progression remains controversial. Prostate cancer with neuroendocrine differentiation was usually considered as AR-null with resistance to the AR-directed interventions (14–16). A previous study revealed that AR and the downstream of pathway are negatively correlated with NE activity in CRPC (14). However, a study in 2019 stratified CRPC into five molecular categories according to the expression of well-characterized AR and NE genes (15). One of these categories is identified as amphicrine tumors, composed of cells co-expressing AR and NE genes. Several studies observed a co-expression area of AR and NE markers in the pathological samples of prostate cancer (15, 17, 18). Another multi-institutional clinical trial also reported cases of retained AR expression and activity in prostate cancer with neuroendocrine differentiation, supporting the clinical value of further investigation in these AR/NE double-positive prostate cancer (6). Interestingly, a recent study investigated the role of AR reprogramming in regulating the lineage plasticity of prostate cancer (18). The results indicated that while the presence of AR promotes the initiation of neuroendocrine differentiation in enzalutamide-sensitive CRPC, the loss of AR drives further neuroendocrine differentiation when it becomes enzalutamide resistant. Therefore, AR may play biphasic roles in different stages of neuroendocrine differentiation in prostate cancer, and the molecular characteristic of AR during the progression of neuroendocrine differentiation would potentially guide the clinical therapeutic strategy and future investigations.

The aim of our work was to shed further light on a better understanding of AR status in prostate cancer with neuroendocrine differentiation. We combined immunostaining, bulk RNA sequencing, and single-cell transcriptome sequencing to assess the AR status in prostate cancer with neuroendocrine differentiation. The exploration of several online bulk RNA sequencing datasets indicated the existence of AR^HIGH^/NE^HIGH^ prostate cancer and revealed that these double-high cases are majorly present in CRPC with a less neuroendocrine-transdifferentiated state. We then reviewed all pathology-confirmed prostate cancer cases with available immunohistochemistry reports in Ren Ji and Xinhua Hospital from 2009 to 2021. Of 8,194 cases, 2.3% (*n* = 189) showed any one of the NE markers (CHGA, SYP, and NCAM1) being positive in at least 5% of epithelial cells, among which 81.0% (*n* = 153) showed AR positive and 19.0% (*n* = 36) showed AR negative. The clinicopathological evaluation showed that a patient with AR loss tumors associated with adverse clinical stages, indicating a trade-off between AR and advanced disease in neuroendocrine differentiation. Using multiplex immuno-fluorescence staining, we observed the co-localization of AR and NE markers in prostate cancer cells. In addition, data mining of single-cell transcriptome further confirmed the existence of AR^HIGH^/NE^HIGH^ prostate cancer cells in castration-resistant samples and suggested that AR still exerts its androgen response and anti-apoptotic effect in these double-high cells. Thus, our study provides a better understanding of AR signaling in cellular plasticity in prostate cancer with neuroendocrine differentiation and allows new insights into therapeutic development.

## Materials and methods

### Study cohort and clinical data collection

We retrospectively reviewed all 8,194 patients with pathologically proven prostate cancer and available immunohistochemistry reports who have visited the Department of Urology in Ren Ji and Xinhua Hospital for a period of 13 years from 2009 to 2021. The reviewed clinical data obtained from electronic records included age, Gleason Score, clinical TNM stage, hormone treatment status, and serum prostate-specific antigen (PSA) level. These clinical data had been reviewed by urologists (RS, KS, and MY). The clinical TNM stage was determined according to the American Joint Committee on Cancer staging system. The PSA serum level depended on the most recent time before sample acquisition. The pathological samples had been reviewed by a pathologist (ZJ) and were classified morphologically according to WHO (9). No adenocarcinoma with Paneth cell neuroendocrine differentiation and carcinoid tumor were reported. **Table 1** shows a summary of the patient baseline and clinicopathological characteristics.

**Table 1:** Summary of NE markers status in 189 prostate cancer cases with neuroendocrine differentiation.

NE markers	CHGA (%)	SYP (%)	NCAM1 (%)
**Available**	97	169	73
Positive	61 (62.9)	151 (89.3)	35 (47.9)
Negative	36 (37.1)	18 (10.7)	38 (52.1)
**Not available**	92	20	116

### Statistical analysis

Analysis was performed by using SPSS Statistics v24 (IBM, Chicago, IL, USA). Categorical variables between groups were tested by using chi-square test or two-tailed Fisher’s exact test. Continuous variables between groups were tested by using unpaired t-test. The relationships between continuous variables were tested by Pearson’s correlation coefficient. P < 0.05 was considered statistically significant (**P < 0.01, ***P < 0.001).

### Immunohistochemistry staining

Patient prostate tissue samples derived from radical surgery or puncture biopsy were made into 4-μm formalin-fixed paraffin-embedded sections on adhesive-coated glass slides. Antigen retrieval was performed with EDTA solution (pH = 9.0) at 95°C for 20 min after deparaffinizing the formalin-fixed paraffin-embedded sections. Human sections were blocked by 10% horse serum at room temperature for 1 h and then stained with primary antibodies targeting AR (1:100, Rabbit, RMA-0807, MXB biotechnologies), CHGA (1:100, Rabbit, RMA-0548, MXB biotechnologies), SYP (1:100, Mouse, MAB-0742, MXB biotechnologies), and NCAM1 (MAB-0743, Mouse, MXB biotechnologies) at 4°C overnight. Secondary anti-rabbit/mouse antibody (Jackson ImmunoResearch Laboratories) was diluted at 1:500, and the sections were incubated at room temperature for 1 h.

### Multiplex immunofluorescence staining

Multiplex immunofluorescence staining was also performed on 4-μm formalin-fixed paraffin-embedded sections. Antigen retrieval was performed with EDTA solution (pH = 9.0) at 95°C for 20 min after deparaffinizing the formalin-fixed paraffin-embedded sections. Human sections were blocked by 10% horse serum with 0.3 M glycine at room temperature for 1 h and then stained with primary antibodies targeting AR (1:200, rabbit, ab133273, abcam) at 4°C overnight. Secondary anti-rabbit antibody (Jackson ImmunoResearch Laboratories) was diluted at 1:2,000, and the sections were incubated at room temperature for 1 h. Horseradish peroxidase (HRP) was permanently fluorescence-labeled in secondary antibody by TYR-Cy3 (Recordbio) at room temperature for 10 min. Then, antigen retrieval was performed again with EDTA solution (pH = 9.0) at 95°C for 20 min. Human sections were blocked by 10% horse serum with 0.3 M glycine at room temperature for 1 h and then stained with primary antibodies targeting SYP (1:1,000, rabbit, ab32127, abcam) at 4°C overnight. Secondary anti-rabbit antibody (Jackson ImmunoResearch Laboratories) was diluted at 1:2,000, and the sections were incubated at room temperature for 1 h. HRP was permanently fluorescence-labeled in secondary antibody by TYR-488 (RecordBio) at room temperature for 10 min. DAPI (D9542, Sigma), diluted 1:1,000 in PBS, was used for nuclear staining.

### Bulk RNA sequencing analysis and AR/NE signature score

Bulk RNA sequencing data of prostate cancer were collected from online datasets The Cancer Genome Atlas (TCGA) (19), SMMU (20), DKFZ (21), GSE77930 (22), GSE147250 (23), SU2C/PCF (24), and Beltran 2016 (14). According to a previous study (16), AR/NE signature score was calculated in R by using ssGSEA method in Gene Set Variation Analysis package v1.40.1 (25), using the mRNA expression of 10 AR signature genes (*KLK3*, *KLK2*, *STEAP4*, *TMPRSS2*, *FKBP5*, *ALDH1A3*, *NKX3-1*, *PPAP2A*, *PMEPA1*, and *PART1*) and 10 NE signature genes (*CHGA*, *SYP*, *CHGB*, *ENO2*, *ELAVL4*, *NKX2-1*, *SCG3*, *CHRNB2*, *SCN3A*, and *PCSK1*) as input. The ssGSEA method integrated the difference of empirical cumulative distribution functions between the genes inside and outside the gene set, resulting in a signature score that reflects the absolute enrichment degree of the given gene set in each sample (25).

### Single-cell transcriptome sequencing analysis

The single-cell transcriptome cohort consists of six CRPC patient samples from our center which has been published previously (GSE137829) (26). The sequencing coverage and quality statistics for each sample could be found in a previous study (26). The raw gene expression matrix obtained after raw data pre-processing was analyzed in R by Seurat package v4.0.2. Each cell with 500–7,000 detected genes, <10% mitochondrial gene expression, and <3% red blood cell gene expression was filtered after Seurat-based quality control. Epithelial cells were extracted according to the expression level of well-established marker genes *EPCAM*, *CDH1*, *KRT5*, *KRT8*, *KRT 14*, and *KRT18*. Uniform Manifold Approximation and Projection was used to visualize each epithelial cell cluster. Differentially expressed genes between two cell groups were determined by using FindMarkers function in Seurat package. For pathway enrichment assessment, only differentially expressed genes with fold-change >1.2 and *P <*0.01 were input into GSEA function in clusterProfiler package v3.18.1.

## Results

### Comparative analysis of AR/NE-associated genes in bulk prostate cancer transcriptome datasets revealed the existence of AR^HIGH^/NE^HIGH^ cases

To investigate the AR status in prostate cancer with neuroendocrine differentiation, we first analyzed the bulk RNA sequencing datasets consisting of hormone-naïve prostate cancer cohorts (TCGA (19), SMMU (20), and DKFZ (21)) and castration-resistant prostate cancer cohorts (GSE77930 (22), GSE147250 (23), SU2C/PCF (24), and Beltran 2016 (14)).

**Figure 1:**
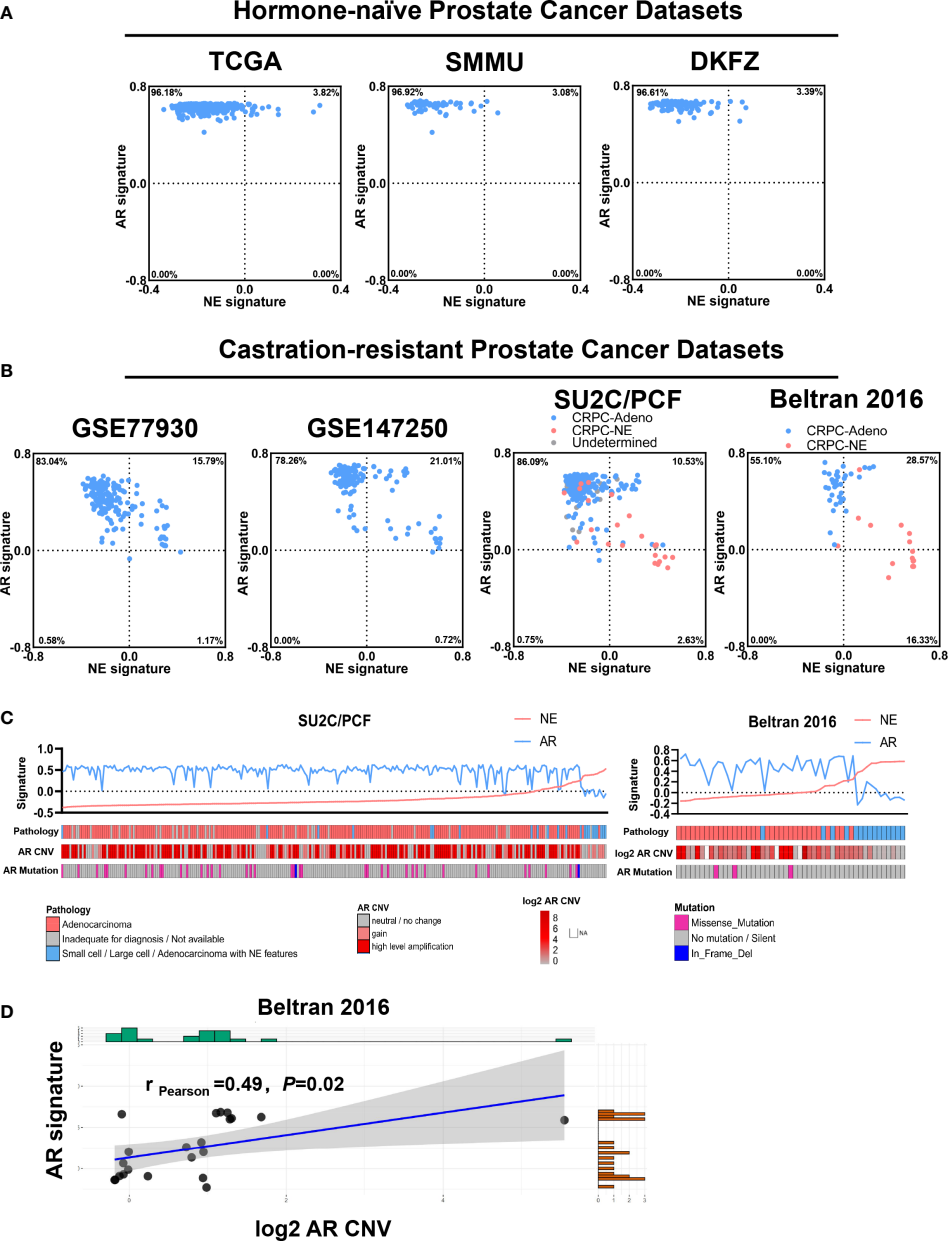
Comparative analysis of androgen receptor (AR)/neuroendocrine (NE)-associated genes in bulk prostate cancer transcriptome datasets. **(A)** Scatterplot showing the calculated AR and NE signature in hormone-naïve prostate cancer cohorts from The Cancer Genome Atlas (*n* = 498), SMMU (*n* = 65), and DKFZ (*n* = 118). The blue points indicate pathology-confirmed adenocarcinoma without neuroendocrine features, and the red points indicate pathology-confirmed prostate cancer with neuroendocrine features. **(B)** Scatterplot showing the calculated AR and NE signatures in castration-resistant prostate cancer cohort from GSE77930 (*n* = 171), GSE147250 (*n* = 138), SU2C/PCF (*n* = 266), and Beltran 2016 (*n* = 49). The blue points indicate pathology-confirmed adenocarcinoma without neuroendocrine features, the red points indicate pathology-confirmed prostate cancer with neuroendocrine features, and the gray points indicate prostate cancer with undetermined pathology classification. **(C)** Top: plot reporting NE signature (red line) and AR signature (blue line) across cases in the SU2C/PCF and the Beltran 2016 cohorts. Cases were ordered by increasing the values of NE signature within each cohort. Bottom: annotations or heat map showing the pathology classification, AR copy number variation, and AR mutation status of each case. **(D)** Correlation between AR signature score and log2 AR copy number variation across the NE^HIGH^ cases from the Beltran 2016 cohort. The shadow area indicates the 95% confidence interval. Pearson’s correlation coefficient and *P*-value are shown on the plot.

We calculated the AR and NE scores according to 10 AR signature genes (*KLK3*, *KLK2*, *STEAP4*, *TMPRSS2*, *FKBP5*, *ALDH1A3*, *NKX3-1*, *PPAP2A*, *PMEPA1*, and *PART1*) and 10 NE signature genes (*CHGA*, *SYP*, *CHGB*, *ENO2*, *ELAVL4*, *NKX2-1*, *SCG3*, *CHRNB2*, *SCN3A*, and *PCSK1*), respectively, as described previously (16) (**Figures 1A, B**). The 10-gene AR or NE signatures—which represented the absolute degree of AR or NE pathway activity in each sample—were highly concordant with other gene sets indicative of AR or NE pathway activity, respectively (**Supplementary Figure S1**) (14, 16, 27). As shown in **Figures 1A, B** the patient samples were categorized into four quadrants based on AR and NE signatures, and it is no surprise that all the cases with hormone-naïve prostate cancer exhibited a high level of AR signaling. Among these cases, a small and comparable proportion gained a high level of NE signaling, termed AR^HIGH^NE^HIGH^: 3.82% in TCGA, 3.08% in SMMU, and 3.39% in DKFZ. These NE and AR double-high proportion was largely increased in the datasets with a castration-resistant cohort: 15.79% in GSE 77930, 21.01% in GSE147250, 10.53% in SU2C/PCF, and 28.57% in Beltran 2016.

We further analyzed two CRPC datasets—SU2C/PCF and Beltran 2016—which contain both adenocarcinomas (CRPC-Adeno) and prostate cancer with neuroendocrine features (CRPC-NE). When focusing on CRPC-NE, we observed that the AR^HIGH^ and the AR^LOW^ cases approximately account for half and half of the NE^HIGH^ tumors, indicating that some tumors still maintain a relatively high level of AR signaling even with pathologically positive neuroendocrine markers (**Figure 1B**). We also noticed that all the AR^LOW^/NE^HIGH^ cases belong to CRPC-NE, indicating that the downregulation of AR signaling as well as the upregulation of NE signaling mostly happened when the pathological neuroendocrine features arose.

We next examined the genomic-level AR mutation and copy number variation (CNV) in these two CRPC datasets (**Figure 1C**). The results supported the correlation between the AR score and AR amplification copy number in NE^HIGH^ cases: in the SU2C/PCF dataset, a high level amplification of AR copy number was detected in 53.6% of the AR^HIGH^/NE^HIGH^ cases but none in the AR^LOW^/NE^HIGH^ cases (*P* = 0.031, two-tailed Fisher’s exact test); in the Beltran 2016 dataset, the AR signature positively correlated with the AR CNV (Pearson’s correlation coefficient = 0.49, *P* = 0.02, **Figure 1D**). These findings indicated that the alternation of AR copy number at the genomic level may be responsible for the upregulation of the AR signaling in NE^HIGH^ cases.

These data altogether suggest that the castration-resistant cohorts contain a larger proportion of cases with high NE signature than hormone-naïve cohorts, and most importantly, AR^HIGH^/NE^HIGH^ cases are present in all cohorts, especially in castration-resistant cohorts with a less differentiated state—which presented high NE signaling but no pathological neuroendocrine features yet; when comes to the tumors with a more differentiated state that presented both high NE signaling and pathological neuroendocrine features, the AR signaling would decrease accompanied with the upregulation of NE signaling.

### Clinicopathological features indicated that loss of AR was positively correlated with adverse clinical stage in prostate cancer with neuroendocrine differentiation

The abovementioned results prompted us to assess the clinical correlation of AR status in prostate cancer with neuroendocrine differentiation. We then retrospectively reviewed 8,194 pathology-confirmed primary prostate cancer cases with available immunohistochemistry reports in two large clinical centers from 2009 to 2021. Among these 8,194 cases, 2.3% (*n* = 189) showed at least one of the NE markers (CHGA, SYP, and NCAM1) being positive in at least 5% of epithelial cells. **Table 1** shows a summary of the three NE markers’ expression status in these 189 cases. Consistent with a previous study, which identified SYP as the highest sensitive marker in neuroendocrine carcinoma of the genitourinary tract, including prostate cancer, our results also showed that SYP had the highest positive rate among the three NE markers in prostate cancer with neuroendocrine differentiation (28). Within these NE-positive cases, AR was positive (AR+) in 81.0% cases (*n* = 153) and negative (AR-) in 19% cases (*n* = 36). Moreover, the NE positive cases showed a slightly higher incidence of AR- than NE-negative cases (19.0 *vs*. 13.3%, *P* = 0.021, chi-square test; **Table 2**).

**Table 2:** Summary of AR status in 8194 pathology-confirmed prostate cancer cases.

	NE-negative (%)	NE-positive (%)	Total	*P*
**No. of patients**	8005 (97.7)	189 (2.3)	8194	
**Median age (Range)**	69.3 (32-92)	70 (36-96)	70 (32-96)	0.557
**AR status**				
Positive	6943 (86.7)	153 (81.0)	7096	0.021
Negative	1062 (13.3)	36 (19.0)	1098		

We focused on these 189 prostate cancer cases with neuroendocrine differentiation and summarized the baseline and clinicopathological characteristics in **Table 3**. PSA was positive in 94.8% of AR+ cases and only in 16.7% of AR- cases, indicating that the expression of AR at the tissue level was highly concordant with the activation of the AR pathway. The median patient age groups at baseline showed no difference between the AR+ cases and AR- cases (*P* = 0.844, chi-square test). Though 39.2% (*n* = 74) cases had received hormone treatment, none of these cases met the diagnostic criteria of CRPC according to the EAU-EANM-ESTRO-ESUR-SIOG guidelines on prostate cancer at the time of tissue acquisition (29). The proportion of hormone-treated cases was similar between the AR+ and AR- cases (38.6 *vs*. 41.7%, *P* = 0.731, chi-square test). Within the 189 cases, AR- cases were positively correlated with higher T stage (86.1 *vs*. 40.5%, *P* < 0.001, chi-square test), lymph node metastasis rate (69.4 *vs*. 24.2%, *P* < 0.001, chi-square test), and distant metastasis rate (63.9 *vs*. 19.6%, *P* < 0.001, chi-square test) than AR+ cases. Gleason Score is another widely used index to evaluate the malignancy of prostate cancer, but it could not be assigned if areas of tumor lack glandular differentiation due to neuroendocrine transdifferentiation or treatment-induced reaction. AR- cases showed a higher rate of patients with non-applicable Gleason Score than AR+ cases (52.8 *vs*. 10.5%, *P* < 0.001, chi-square test) because of the higher proportion of small or large cell carcinoma (55.6 *vs*. 2.0%, *P* < 0.001, chi-square test). On the other hand, within all of the rest of the cases with applicable Gleason Score, AR- cases showed a higher rate of patients with Gleason Score 9 to 10 than AR+ cases (70.6 *vs*. 30.7%, *P* = 0.001, chi-square test), indicating the much poorer differentiation in tumors from AR- cases. AR+ cases showed a higher median serum PSA level than the AR- cases (9.69 *vs*. 4.25, *P* = 0.684, unpaired *t*-test), indicating the retained function of AR signaling.

**Table 3:** Baseline and clinicopathological characteristics of prostate cancer with neuroendocrine differentiation (n=189).

AR status		AR+ (%)	AR- (%)	Total	*P*
**No. of patients**		153(81.0)	36(19.0)	189	
**PSA status**					<0.001
Positive		145 (94.8)	6 (16.7)	151	
Negative		8 (5.2)	30 (83.3)	38	
**Age (years)**					0.844
<65		32(20.9)	7(19.4)	39	
>=65		121(79.1)	29(80.6)	150	
**Gleason Score**					<0.001
8-Jul		95(62.1)	5(13.9)	100	
10-Sep		42(27.5)	12(33.3)	54	
Non-applicable		16(10.5)	19(52.8)	35	
**Clinical TNM stage**					
T					<0.001
T1-2		91(59.5)	5(13.9)	96	
T3-4		62(40.5)	31(86.1)	93	
N					<0.001
N0		116(75.8)	11(30.6)	127	
N1		37(24.2)	25(69.4)	62	
M					<0.001
M0		123(80.4)	13(36.1)	136	
M1		30(19.6)	23(63.9)	53	
**Median serum PSA**(**ng/mL**)		9.69	4.25	9.1	0.684
**Hormone treatment status**					0.731
Hormone-naïve		94(61.4)	21(58.3)	115	
Hormone-treated		59(38.6)	15(41.7)	74	
**Sample resource**					<0.001
Radical surgery		86(56.2)	7(19.4)	93	
Puncture biopsy		67(43.8)	29(80.6)	96	
**Morphological classification**					<0.001
Usual prostate adenocarcinoma with neuroendocrine differentiation		146(95.4)	15(41.7)	161	
Small cell carcinoma		3(2.0)	19(52.8)	22	
Large cell neuroendocrine carcinoma		0	1(2.8)	1	
Mixed (small or large cell) neuroendocrine					
		4(2.6)	1(2.8)	5	

We next grouped these 189 cases by hormone treatment status and further analyzed the clinical characteristics within each subgroup. The results were similar in both hormone-naïve and hormone-treated settings: AR- cases were associated with higher TNM stages and poorer Gleason Score in comparison to AR+ cases (**Supplementary Tables S1, S2**). Our findings indicated that, in pathology-confirmed prostate cancer with neuroendocrine differentiation, AR still expressed and functioned in a considerable part of the tumor; loss of AR expression was associated with adverse clinical stage and poor cancer cell differentiation.

### Immunohistochemistry staining and multiplex immunofluorescence staining reveal the localization of AR and NE markers in prostate cancer with neuroendocrine differentiation

Next, we asked whether the expression of AR and NE markers could co-exist in the same area of tissue. To address this question, we performed immunohistochemistry staining of AR, PSA, and different NE markers in serial pathological sections of 189 prostate cancer patients with neuroendocrine differentiation. **Table 1** shows a summary of the overall expression status of the three NE markers, including CHGA, NCAM1, and SYP.

**Figure 2:**
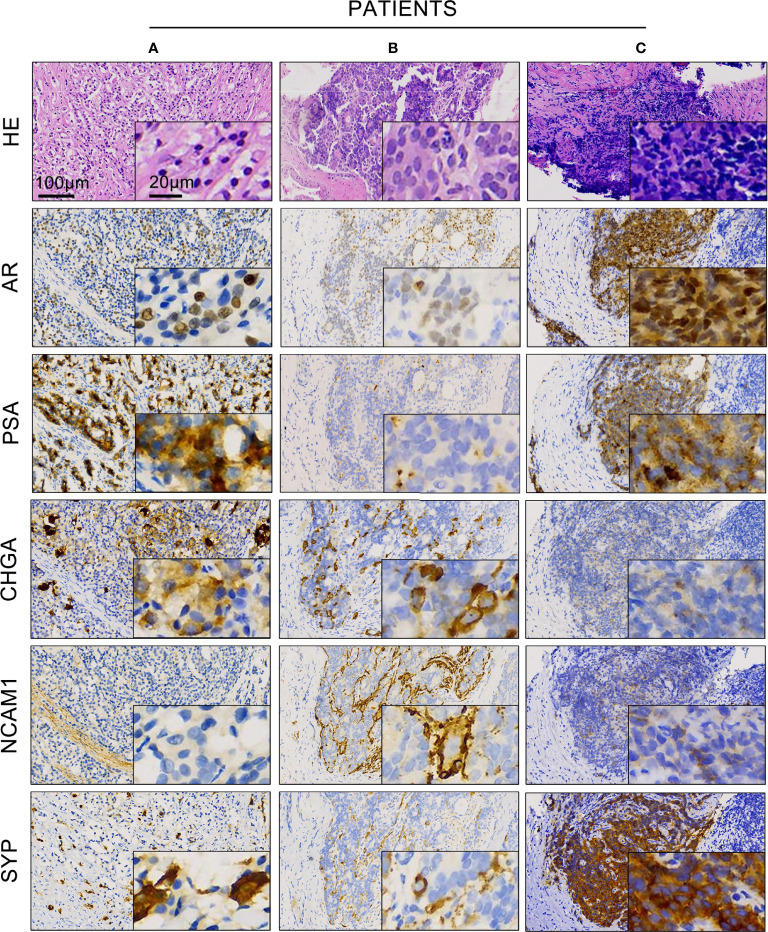
Expression of androgen receptor (AR), prostate-specific antigen (PSA), and different neuroendocrine markers in prostate cancer with neuroendocrine differentiation. Representative hematoxylin and eosin and immunohistochemistry staining showed the expression of AR, PSA, CHGA, NCAM1, and SYP in prostate cancer patient samples **(A–C)** of the AR+ cases.

As shown in **Figure 2**, patients A and B were both diagnosed as usual prostate adenocarcinoma with neuroendocrine differentiation. The expressions of AR and PSA were both strong in patient A but occasional in patient B. Patient C was diagnosed as small cell prostate carcinoma with a strong expression of both AR and PSA. Although the expression of CHGA, NCAM1, and SYP varied among these three individuals, all of them showed a co-expression of AR and NE markers in the same areas of tumor sites.

**Figure 3:**
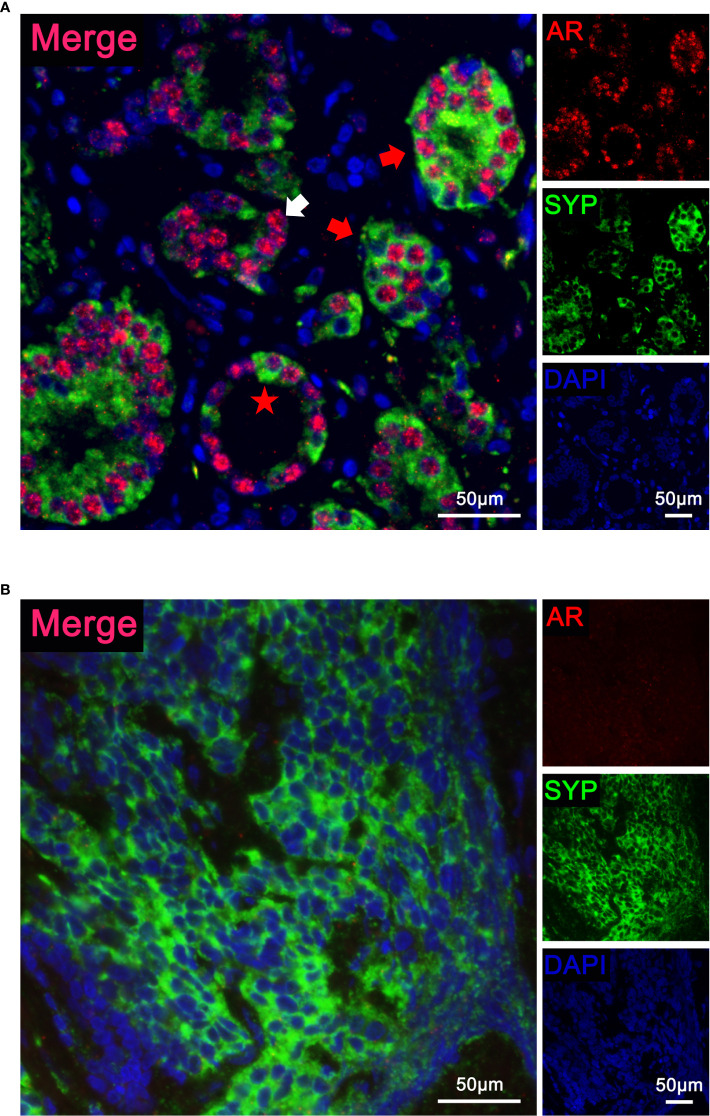
Localization of androgen receptor (AR) and synaptophysin (SYP) in prostate cancer with neuroendocrine differentiation. **(A)** Representative immunofluorescence images of a hormone-naïve AR+ case with Gleason Score 7 simultaneously stained with a three-color multiplex panel containing DAPI (blue), AR (red), and SYP (green). The white arrowhead indicates malignant tumor tissue with AR expression only. The red arrowheads indicate malignant tumor tissue with both AR and SYP expression. The red star indicates an AR and SYP double-positive cell in adjacent premalignant prostate lesion. **(B)** Representative immunofluorescence images of a hormone-naïve AR- case with non-applicable Gleason Score simultaneously stained with a three-color multiplex panel containing DAPI (blue), AR (red), and SYP (green). The tumor cells presented small cell morphology with high malignancy and showed positive SYP.

We further asked whether AR and NE markers can co-localize in the same cell. Using multiplex immunofluorescence staining, we detected AR and SYP simultaneously in tumor samples consisting of both premalignant prostate lesions and malignant tumor tissues. **Figure 3A** presented a hormone-naïve AR+ case with Gleason Score 7. The localization pattern of AR and SYP could be various: the white arrowhead indicates a malignant prostate tissue that consisted of epithelial cells expressing AR only, and the red arrowheads indicate malignant prostate tissues that consisted of epithelial cells expressing both AR and SYP. Notably, we also observed AR and SYP double-positive cells (red star) that were present in the adjacent premalignant prostate lesion. Thus, multiplex immunofluorescence staining demonstrated that AR and NE markers can co-localize in the same prostate cancer cell. **Figure 3B** presented another hormone-naïve AR- case with non-applicable Gleason Score. The tumor cells showed small cell morphology with high malignancy and expressed SYP only.

### Comparative analysis of AR/NE-associated genes in single-cell transcriptome from six CRPC patients

**Figure 4:**
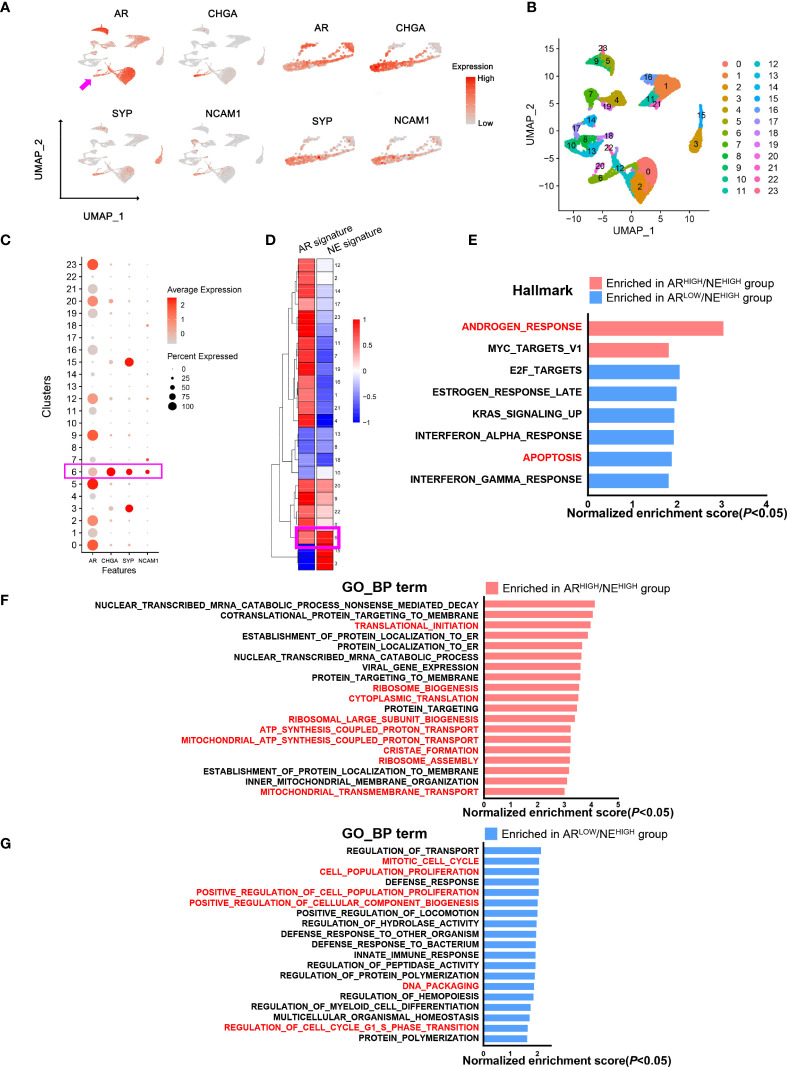
Comparative analysis of androgen receptor (AR)/neuroendocrine (NE)-associated genes in a single-cell transcriptome from prostate cancer patients. **(A)** Uniform Manifold Approximation and Projection plot showing an overlap of AR and NE marker expressions in prostate epithelial cells. **(B)** Uniform Manifold Approximation and Projection plot showing the cell clustering of prostate epithelial cells. **(C)** Dot plot showing the expression status of AR and NE markers in different cell clusters. **(D)** Heat map showing AR and NE signature scores in different cell clusters. **(E)** Pathway enrichment assessment of differentially expressed genes showing enriched Hallmark pathways in either AR^HIGH^/NE^HIGH^ cells or AR^LOW^/NE^HIGH^ cells (*P* < 0.05; false discovery rate (FDR), <25%). **(F)** Pathway enrichment assessment of differentially expressed genes showing the top enriched Gene Ontology biological process pathways in AR^HIGH^/NE^HIGH^ cells (*P* < 0.05, FDR < 25%). **(G)** Pathway enrichment assessment of differentially expressed genes showing the top enriched Gene Ontology biological process pathways in AR^LOW^/NE^HIGH^ cells (*P* < 0.05, FDR < 25%).

Finally, to further characterize the potential function of AR in neuroendocrine prostate cancer cells, we analyzed the single-cell transcriptome data of six CRPC patient samples previously reported by our center (GSE137829) (26). We extracted epithelial cells according to well-established marker genes *EPCAM*, *CDH1*, *KRT5*, *KRT8*, *KRT 14*, and *KRT18.* We found a cell cluster with an overlap expression of *AR*, *CHGA*, *SYP*, and *NCAM1* (**Figure 4A**). Through unbiased cell clustering, we confirmed the high expression level of these genes in epithelial cluster 6 which also presented high AR and NE signature scores (**Figures 4B–D**). This result further confirmed the existence of AR^HIGH^/NE^HIGH^ prostate cancer in the single-cell level. In order to explore the role of AR in NE^HIGH^ prostate cancer cells, we extracted cells in NE^HIGH^ clusters (clusters 0, 3, 6, 9, 15, 20, and 22) and divided them into the AR^HIGH^/NE^HIGH^ and AR^LOW^/NE^HIGH^ groups according to the expression level of *AR*. Within the cells from NE^HIGH^ clusters, the median expression level of AR was first determined, and cells with AR expression higher than the median level were attributed to the AR^HIGH^/NE^HIGH^ group, while the rest were attributed to the AR^LOW^/NE^HIGH^ group. We identified differentially expressed genes between these two groups and performed a pathway enrichment assessment. The results showed top-enriched Hallmark and Gene Ontology Biological Process pathways (*P* < 0.05, false discovery rate <25%) in the AR^HIGH^/NE^HIGH^ or AR^LOW^/NE^HIGH^ group (**Figures 4E–G**). The androgen response pathway was enriched in the AR^HIGH^/NE^HIGH^ group, while the apoptosis pathway was enriched in the AR^LOW^/NE^HIGH^ group, which further support the concordance of AR expression and the activation of the AR pathway found in our clinical cohort (**Figure 4E**). Consistent with the abovementioned analysis of PSA in tumor samples, these results indicated that AR still exert its function of androgen response and anti-apoptosis in AR^HIGH^/NE^HIGH^ cells. Notably, though AR^HIGH^/NE^HIGH^ cells present a higher level of metabolism activity (**Figure 4F**), AR^LOW^/NE^HIGH^ cells still have a higher proliferation potential as determined by enriched pathways associated with cell cycle and cell proliferation (**Figure 4G**), corresponding to the higher malignancy of AR- prostate cancer with neuroendocrine differentiation compared with AR+.

## Discussion

By using immunostaining and bulk and single-cell transcriptome sequencing, we analyzed the AR status in prostate cancer with neuroendocrine feature to characterize the role of AR and AR signaling in neuroendocrine differentiation. From the multiple bulk RNA sequencing datasets, we found prostate cancer samples with simultaneously high AR and NE signature scores in all of these datasets. The percentage of AR^HIGH^/NE^HIGH^ cases rises in castration-resistant prostate cancer cohorts than in hormone-naïve prostate cancer cohorts. Consistently, other groups also reported the co-expression of AR and NE genes in castration-resistant prostate cancer by using both molecular profiling and histological assessments (15). Notably, in the SU2C/PCF and Beltran 2016 cohorts, which include castration-resistant tumors that are pathologically characterized as adenocarcinoma with or without neuroendocrine features, several cases with a high level of NE signaling and negative AR signaling had developed a neuroendocrine feature in pathological morphology, which represent a more neuroendocrine-differentiated state. It is known that AR signaling is upregulated in prostate adenocarcinoma, including hormone-naïve and castration-resistant adenocarcinoma, and we further concluded that AR signaling maintains a high level in a less neuroendocrine-differentiated state that presented high NE signaling only but no pathological neuroendocrine features yet. On the other hand, though the overlap between AR and NE exists, they would separate and go their own way in a more differentiated state.

We further confirm the co-expression of AR and NE markers in the samples of prostate cancer with neuroendocrine differentiation from our cohort by using immunohistochemistry staining. We retrospectively reviewed all 8,194 prostate cancer cases with available immunohistochemistry reports and found 2.3% (*n* = 189) with any one of the NE markers (CHGA, SYP, and NCAM1) being positive in at least 5% of epithelial cells. Moreover, 81.0% (*n* = 153) of 189 cases showed AR+. By using serial pathological sections, we observed positive-stained AR and representative NE markers in the same area of tissue (**Figure 2**). Other groups also found a co-localization area of AR and NE markers, especially SYP in hormone-treated prostate cancer samples (17, 18). To provide additional evidence for the existence of AR and NE double-positive cells, we performed multiplex fluorescence staining and observed the co-localization of AR and SYP in the same prostate cancer cells (**Figure 3**). These results confirm that AR is still expressed in some tissues of prostate cancer with neuroendocrine differentiation, even overlapping with NE markers in some special cells.

We investigated the clinical features of AR+ *versus* AR- cases in 189 patients with the neuroendocrine features from our cohort and found that loss of AR was significantly correlated with advanced TNM staging and a higher level of Gleason Score (**Table 3**). To explore the molecular events in AR and NE double-high cell population, we applied unbiased single-cell RNA transcriptome analysis with six CRPC patients and identified a cell cluster that expressed both AR and NE markers. We applied pathway enrichment analysis in the NE^HIGH^ clusters and found that the AR^HIGH^/NE^HIGH^ cells may have a higher level of androgen response, anti-apoptotic effect, and metabolic activity compared with the AR^LOW/^NE^HIGH^ cells. On the other hand, the AR^LOW/^NE^HIGH^ cells may have a higher proliferation potential. This is consistent with a growing appreciation of the highly aggressive role of neuroendocrine differentiation with gradual loss of AR in prostate cancer progression.

In summary, our transcriptome profile analysis, immunohistochemistry staining, and multiplex fluorescence staining together demonstrate the existence of AR and NE double-high prostate cancer. Our study also provides a deeper understanding of the AR activity in the neuroendocrine transition of prostate cancer cells and allows future implications for anti-tumor therapies.

## Data availability statement

The original contributions presented in the study are included in the article/**Supplementary Material**. Further inquiries can be directed to the corresponding authors.

## Ethics statement

This study was reviewed and approved by the Committee for Ethics of Ren Ji Hospital and the Committee for Ethics of Xinhua Hospital. The patients/participants provided their written informed consent to participate in this study.

## Author contributions

QW: conceptualization, methodology, writing—original draft, writing—review and editing, supervision, project administration, and funding acquisition. WX: conceptualization, resources, supervision, project administration, and funding acquisition. RS: conceptualization, methodology, validation, formal analysis, investigation, data curation, writing—original draft, and visualization. LC and ZJ: conceptualization, methodology, software, validation, formal analysis, investigation, and data curation. MY, WZ, ZM, YJ, KS, ZX, and JQ: methodology, validation, formal analysis, investigation, and data curation. All authors contributed to the article and approved the submitted version.

## Funding

This work was supported by National Natural Science Foundation of China (No 82172868, 81972578, 82072847, 81772742), Science and Technology Commission of Shanghai Municipality (19XD1402300), Shanghai Municipal Health Commission (2019LJ11, 2020CXJQ03), Shanghai Shenkang Hospital Development Center (SHDC2020CR6008, 16CR3049A), the Program for Professor of Special Appointment (Eastern Scholar) at Shanghai Institutions of Higher Learning (TP2017029); Shanghai Municipal Education Commission-Gaofeng Clinical Medicine Grant Support (20191906, 20171912, 20152215).

## Acknowledgments

We are grateful to the patients and their families who contributed to this research. We thank Lucky Wong for discussion of this study.

## Conflict of interest

The authors declare that the research was conducted in the absence of any commercial or financial relationships that could be construed as a potential conflict of interest.

## Publisher’s note

All claims expressed in this article are solely those of the authors and do not necessarily represent those of their affiliated organizations, or those of the publisher, the editors and the reviewers. Any product that may be evaluated in this article, or claim that may be made by its manufacturer, is not guaranteed or endorsed by the publisher.
