# Supplementary figures and images for: Comprehensive analysis of androgen receptor status in prostate cancer with neuroendocrine differentiation
Source: Front Oncol. 2022 Aug 9;12:955166. doi: 10.3389/fonc.2022.955166 (PMC9413533; doi:10.3389/fonc.2022.955166)

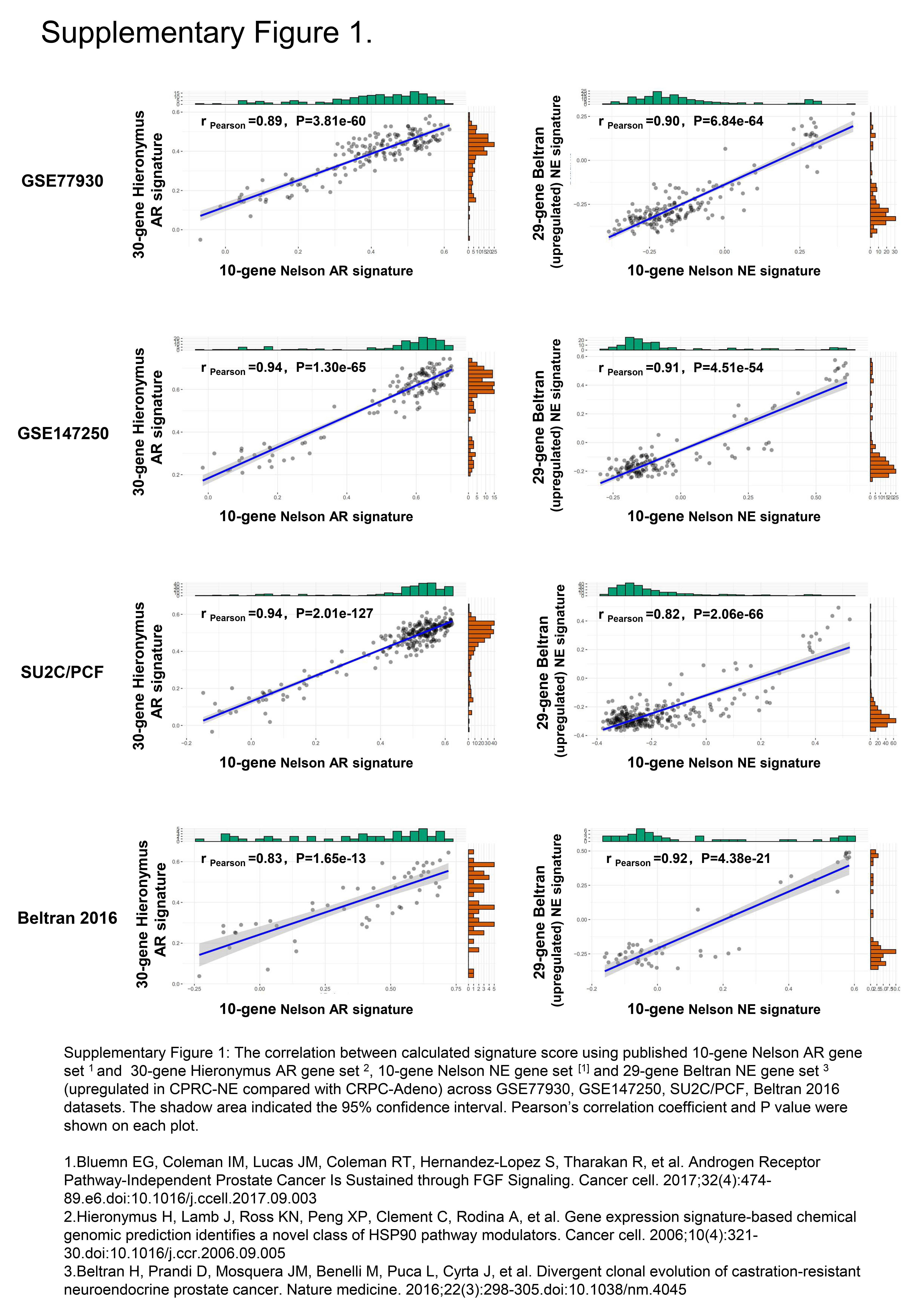

Supplement: Supplementary file 1 [file Image_1.jpeg]
