# Supplementary material for: Comprehensive analysis of androgen receptor status in prostate cancer with neuroendocrine differentiation
Source: Front Oncol. 2022 Aug 9;12:955166. doi: 10.3389/fonc.2022.955166 (PMC9413533; doi:10.3389/fonc.2022.955166)
Supplement: Supplementary file 2 [file Table_1.docx]

**Supplementary Table 1.** Baseline and clinicopathological characteristics of hormone-naïve prostate cancer with neuroendocrine differentiation (n=115).

| **AR status** | **AR+ (%)** | **AR- (%)** | | **Total** | **P** |
| --- | --- | --- | --- | --- | --- |
| No. of patients | 94 | 21 | | 115 |  |
| Age (years) |  |  | |  | 0.923 |
| <65 | 17(18.1) | 3(14.3) | | 20 |  |
| >=65 | 77(81.9) | 18(85.7) | | 95 |  |
| Gleason Score |  |  | |  | <0.001 |
| 7-8 | 73(77.7) | 3(14.3) | | 76 |  |
| 9-10 | 18(19.1) | 4(19.0) | | 22 |  |
| Unevaluable | 3(3.2) | 14(66.7) | | 17 |  |
| Clinical TNM stage |  |  | |  |  |
| T |  |  | |  | <0.001 |
| T1-2 | 62(66.0) | 4(19.0) | | 66 |  |
| T3-4 | 32(34.0) | 17(81.0) | | 49 |  |
| N |  |  | |  | <0.001 |
| N0 | 78(83.0) | 9(42.9) | | 87 |  |
| N1 | 16(17.0) | 12(57.1) | | 28 |  |
| M |  |  | <0.001 | | |
| M0 | 81(86.2) | 9(42.9) | | 90 |  |
| M1 | 13(13.8) | 12(57.1) | | 25 |  |
| Median serum PSA（ng/mL） | 10.82 | 4.25 | | 9.47 | 0.685 |
| Sample resource |  |  | |  | 0.002 |
| Radical surgery | 51(54.3) | 3(14.3) | | 54 |  |
| Puncture biopsy | 43(45.7) | 18(85.7) | | 61 |  |
| Morphological classification |  |  | |  | <0.001 |
| Usual prostate adenocarcinoma with neuroendocrine differentiation | 90(95.7) | 8(38.1) | | 98 |  |
| Small cell carcinoma | 0 | 12(57.1) | | 12 |  |
| Large cell neuroendocrine carcinoma | 0 | 1(4.8) | | 1 |  |
| Mixed (small or large cell) neuroendocrine carcinoma—acinar adenocarcinoma | 4(4.3) | 0 | | 4 |  |

**Supplementary Table 2.** Baseline and clinicopathological characteristics of hormone-treated prostate cancer with neuroendocrine differentiation (n=74).

| **AR status** | **AR+ (%)** | **AR- (%)** | | **Total** | **P** |
| --- | --- | --- | --- | --- | --- |
| No. of patients | 59 | 15 | | 74 |  |
| Age (years) |  |  | |  | 0.920 |
| <65 | 15(25.4) | 3(20.0) | | 18 |  |
| >=65 | 44(74.6) | 12(80.0) | | 56 |  |
| Gleason Score |  |  | |  | 0.203 |
| 7-8 | 22(37.3) | 2(13.3) | | 24 |  |
| 9-10 | 24(40.7) | 8(53.3) | | 32 |  |
| Unevaluable | 13(22.0) | 5(33.3) | | 18 |  |
| Clinical TNM stage |  |  | |  |  |
| T |  |  | |  | 0.007 |
| T2 | 29(49.2) | 1(6.7) | | 30 |  |
| T3-4 | 30(50.8) | 14(93.3) | | 44 |  |
| N |  |  | |  | 0.001 |
| N0 | 38(64.4) | 2(13.3) | | 40 |  |
| N1 | 21(35.6) | 13(86.7) | | 34 |  |
| M |  |  | 0.004 | | |
| M0 | 42(71.2) | 4(26.7) | | 46 |  |
| M1 | 17(28.8) | 11(73.3) | | 28 |  |
| Median serum PSA（ng/mL） | 7.05 | 5.03 | | 7.05 | 0.997 |
| Sample resource |  |  | |  | 0.049 |
| Radical surgery | 35(59.3) | 4(26.7) | | 39 |  |
| Puncture biopsy | 24(40.7) | 11(73.3) | | 35 |  |
| Morphological classification |  |  | |  | <0.001 |
| Usual prostate adenocarcinoma with neuroendocrine differentiation | 56(94.9) | 7(46.7) | | 63 |  |
| Small cell carcinoma | 3(5.1) | 7(46.7) | | 10 |  |
| Large cell neuroendocrine carcinoma | 0 | 0 | | 0 |  |
| Mixed (small or large cell) neuroendocrine carcinoma—acinar adenocarcinoma | 0 | 1(6.7) | | 1 |  |
